# Birth prevalence and characteristics of cleft lip and/or palate in dubai health public hospitals, 2018–2025: a retrospective hospital-based study

**DOI:** 10.3389/fdmed.2026.1982859

**Published:** 2026-10-14

**Authors:** Ali Bahmanshiri, Manal Al Halabi, Iyad Hussein, Eman Alnuaimi, Derek Mendonca, Anas Salami

**Affiliations:** 1 Hamdan Bin Mohammed College of Dental Medicine, Mohammed Bin Rashid University of Medicine and Health Sciences, Dubai Health, Dubai, United Arab Emirates; 2 Department of Plastic and Reconstructive Surgery, Al Jalila Children’s Specialty Hospital, Dubai Health, Dubai, United Arab Emirates

**Keywords:** cleft lip, cleft palate, cleft prevalence, congenital anomalies, epidemiology

## Abstract

**Background:**

Population-based data on cleft lip and/or palate (CL/P) in the United Arab Emirates are limited, and no longitudinal estimate is available for Dubai.

**Objective:**

To estimate hospital-based birth prevalence among births recorded in Dubai Health public hospitals during 2018–2025 and describe cleft groups and case characteristics.

**Methods:**

This retrospective study included live births and stillbirths recorded at Dubai Hospital, Latifa Hospital, and Hatta Hospital. The Dubai Health Data & Insights Team identified potential cases through an electronic health record query for International Classification of Diseases, 10th Revision categories Q35–Q37. Investigators reviewed records and mapped documented diagnoses to four mutually exclusive analytical groups. Birth prevalence and exact Poisson 95% confidence intervals (CIs) were calculated per 1,000 total births. Case characteristics were summarized descriptively; temporal change was assessed by Poisson regression with annual births as offset.

**Results:**

Among 46,418 births, 97 CL/P cases were identified, giving a hospital-based birth prevalence of 2.09 per 1,000 total births (95% CI 1.69–2.55). Cleft palate without lip involvement was the most frequent group (48/97, 49.5%; 1.03 per 1,000), followed by cleft lip without palate involvement (22/97, 22.7%; 0.47 per 1,000), unilateral cleft lip and palate or CLP not otherwise specified (19/97, 19.6%; 0.41 per 1,000), and bilateral cleft lip and palate (8/97, 8.2%; 0.17 per 1,000). Annual prevalence ranged from 1.09 to 2.90 per 1,000; no statistically significant linear trend was detected (rate ratio per year 1.07, 95% CI 0.98–1.17; *p* = 0.122). Twenty-nine cases (29.9%) had a documented associated diagnosis or finding. Restriction to the 68 cases without a documented associated diagnosis or finding yielded 1.46 per 1,000 (95% CI 1.14–1.86). Cleft-group comparisons were exploratory and limited by small strata.

**Conclusion:**

This study provides an eight-year baseline for Dubai Health public hospitals, not an emirate-wide estimate. Integrated public- and private-sector surveillance is needed to estimate population-level birth prevalence and plan multidisciplinary care.

## 1 Introduction

Cleft lip and/or palate (CL/P) comprises a heterogeneous group of congenital craniofacial anomalies involving the lip, alveolus, and/or palate. These conditions arise when embryologic facial processes fail to grow or fuse normally during early gestation and are commonly classified into cleft lip (CL), cleft palate (CP), and cleft lip with palate (CLP) phenotypes (1–4). The causal architecture is multifactorial: genetic susceptibility interacts with environmental and maternal influences, including tobacco exposure, selected systemic conditions, nutritional factors, and other pregnancy-related exposures (4–8).

The implications extend well beyond the primary anatomical defect. Affected children may experience feeding and airway difficulties, recurrent otologic disease and hearing impairment, speech and language disorders, dental anomalies, malocclusion, psychosocial burden, and reduced oral-health-related quality of life (9–12). Dental findings can include tooth agenesis or supernumerary teeth, microdontia, delayed or ectopic eruption, enamel defects, and altered morphology of the primary and permanent dentitions (9). CL/P therefore requires coordinated interdisciplinary care from infancy through later childhood, adolescence, and adulthood (13). Reliable epidemiological information is essential for service planning and longitudinal quality improvement.

Reported birth prevalence varies across populations and study designs. Global reviews have shown substantial heterogeneity across phenotypes, geographic regions, ancestries, ascertainment systems, and the inclusion of syndromic cases, stillbirths, or pregnancy terminations (1–3). A 2025 critical review of Arab-country studies reported values ranging from 0.3 to 2.6 per 1,000 live births, with an average of approximately 1.1 per 1,000, but emphasized that many estimates were based on hospital samples with inconsistent definitions and incomplete national surveillance (14). In Qatar, a retrospective study covering approximately 89% of national live births recorded 148 cases among 147,727 live births from 2016 through 2021, corresponding to 1.00 per 1,000 live births (95% CI 0.85–1.18) (15).

Historical UAE evidence is sparse. At Corniche Hospital in Abu Dhabi, Al Talabani et al. reported 13 CL/P cases among 24,233 consecutive live births between 1992 and 1995, equivalent to approximately 0.54 per 1,000 live births (16). A second Abu Dhabi study demonstrated how estimates derived from inpatient birth cohorts and outpatient referral populations can differ because the underlying sampling frames are not equivalent (17). These studies predate contemporary electronic health records and do not provide a Dubai-specific longitudinal estimate.

Recent multicountry research has begun to characterize cleft phenotypes in the region. Alamoudi et al. studied children with non-syndromic cleft lip with or without palate who attended referral centers in seven Middle Eastern and Asian countries, including the UAE, during a one-year recruitment period (18). That study was designed to compare phenotype and severity among patients; it did not provide a birth denominator, a Dubai-specific estimate, or longitudinal surveillance. Accordingly, the birth prevalence and case characteristics of CL/P within Dubai's public hospital system remained unknown.

This study therefore aimed to estimate the hospital-based birth prevalence of CL/P among live births and stillbirths recorded in Dubai Health public hospitals over eight years, from 1 January 2018 through 31 December 2025. Secondary objectives were to describe cleft-group distribution, temporal patterns, sex, gestational age, maternal age, nationality, birth hospital, living status at birth, and documented associated diagnoses or findings. The research question was: what was the hospital-based birth prevalence of CL/P among births recorded in Dubai Health public hospitals during 2018–2025, and how were affected cases distributed by anatomical cleft group and selected demographic, perinatal, and clinical characteristics?.

## 2 Materials and methods

### 2.1 Study design and reporting framework

This retrospective, hospital-based prevalence study used a census of eligible electronic birth records from three public hospitals in Dubai. The report was prepared in accordance with the Strengthening the Reporting of Observational Studies in Epidemiology (STROBE) guidance for cross-sectional studies (19).

### 2.2 Setting

The study was conducted within Dubai Health at Dubai Hospital, Latifa Hospital, and Hatta Hospital. These facilities use the Epic/SALAMA® electronic health record (EHR) system, implemented across the public health network before the study period (20, 21). The observation period extended from 1 January 2018 to 31 December 2025. This period was selected because 2018 was the first full calendar year for which birth records were captured electronically across all three participating facilities, and 31 December 2025 was the end of the last complete calendar year available at extraction in March 2026. The sampling frame included births recorded at these hospitals and should not be interpreted as encompassing all births in the Emirate of Dubai, because private-sector facilities were outside the study network.

### 2.3 Eligibility and case ascertainment

All live births and stillbirths recorded at the participating hospitals from 1 January 2018 through 31 December 2025 were eligible for the denominator. Potential cases were identified through an Epic/SALAMA EHR query for ICD-10 categories Q35–Q37 performed by the Dubai Health Data & Insights Team in March 2026. Eligibility was determined by date of birth within the study period. A cleft diagnosis recorded either during the birth admission or at any subsequent Dubai Health encounter before the March 2026 extraction could identify an eligible case. Births outside the participating hospitals and referrals without a birth record in the participating system were excluded. The initial extract contained 100 rows. The Data & Insights analyst identified three duplicate rows on the basis of repeated patient medical record numbers and removed them, leaving 97 unique patient records. Each medical record number was counted once, irrespective of the number of encounters in which the diagnosis appeared. No additional record was excluded after clinical review.

Case identification used the World Health Organization International Statistical Classification of Diseases and Related Health Problems, 10th Revision (ICD-10) categories Q35–Q37 (22). The codes searched comprised Q35.1 (cleft hard palate), Q35.3 (cleft soft palate), Q35.5 (cleft hard palate with cleft soft palate), Q35.7 (cleft uvula), Q35.9 (cleft palate, unspecified), Q36.0 (cleft lip, bilateral), Q36.1 (cleft lip, median), Q36.9 (cleft lip, unilateral; including cleft lip NOS), Q37.0 (cleft hard palate with bilateral cleft lip), Q37.1 (cleft hard palate with unilateral cleft lip), Q37.2 (cleft soft palate with bilateral cleft lip), Q37.3 (cleft soft palate with unilateral cleft lip), Q37.4 (cleft hard palate with cleft soft palate and bilateral cleft lip), Q37.5 (cleft hard palate with cleft soft palate and unilateral cleft lip), Q37.8 (unspecified cleft palate with bilateral cleft lip), and Q37.9 (unspecified cleft palate with unilateral cleft lip; including cleft palate with cleft lip NOS) (22). The dataset returned by the Dubai Health Data & Insights Team contained the text of the documented clinical diagnosis rather than the electronic ICD-10 code. The principal investigator mapped each documented diagnosis to the corresponding ICD-10 subcategory and analytical cleft group after reviewing the clinical notes and final documented diagnosis. Eight of the 16 searched codes were represented (Supplementary Table S1). In 38 of 97 cases (39.2%), documentation did not support a more anatomically specific category: 11 were Q35.9 and 27 were Q37.8 or Q37.9. Laterality and palatal extent were recorded separately when documented.

### 2.4 Variables and definitions

The primary outcome was CL/P among total births. Cases were grouped into four mutually exclusive anatomical categories: cleft palate without lip involvement (CP), cleft lip without palate involvement (CL), unilateral cleft lip and palate or CLP not otherwise specified (UCLP/CLP-NOS), and bilateral cleft lip and palate (BCLP). Anatomical descriptions were reconciled with the LAHSAL framework where sufficient detail was available (23). Diagnoses confined to the palate (Q35.x) were assigned to CP; diagnoses confined to the lip (Q36.x) to CL; Q37.8 to BCLP; and Q37.9 to UCLP/CLP-NOS. WHO ICD-10 includes both “unspecified cleft palate with unilateral cleft lip” and “cleft palate with cleft lip NOS” under Q37.9; side was retained as unspecified when it was not documented and was not inferred from the code (22). All six records documenting Robin sequence also documented cleft palate without lip involvement and were assigned anatomically to CP; this allocation did not designate them as etiologically isolated, and Robin sequence remained an associated sequence (24). Bilateral cleft lip without palatal involvement (Q36.0) was assigned to CL. Complete and incomplete clefts were eligible when a Q35–Q37 diagnosis was documented and were assigned by the anatomical structures recorded as involved; however, the retrospective free-text data did not permit a reliable complete-vs.-incomplete severity distinction. Each case carried one documented cleft diagnosis, and no adjudication between overlapping codes was required. Submucous cleft palate could not be identified reliably, and alveolar involvement is not separately codable in ICD-10.

Secondary variables were sex recorded at birth, gestational age, maternal age at delivery, nationality, participating hospital, living status at birth, medical history, and documented associated diagnoses or findings. The latter category included a recognized syndrome, chromosomal or molecular entry, sequence, or additional congenital anomaly. Gestational age was grouped as extremely preterm (<28 weeks), very preterm (28–31 weeks), moderately preterm (32–33 weeks), late preterm (34–36 weeks), term (37–41 weeks), or post-term (≥42 weeks) (25). Maternal age was grouped as <30, 30–34, 35–39, or ≥40 years. The ≥35-year threshold was interpreted in the context of current obstetric guidance on pregnancy at age 35 years or older, while retaining the prespecified categories (26). Nationality was the administrative nationality documented in the EHR and denotes citizenship or legal status, not ethnicity or genetic ancestry.

### 2.5 Data collection and quality assurance

Data were obtained under a formal request approved by the Dubai Health Data & Insights Team, which specified ICD-10 categories Q35–Q37 and the study variables. A designated analyst extracted data from Epic/SALAMA and supplied the principal investigator with a password-protected spreadsheet; the password was transmitted separately. The file contained free-text clinical diagnoses, associated conditions, birth hospital, and nationality. The principal investigator coded these data to the study proforma and reviewed the complete Dubai Health record of every identified case, including documentation after the birth admission and before extraction, to confirm eligibility and anatomical cleft characteristics. The cleft phenotype was based on physical-examination findings documented by treating clinicians; imaging was not used to confirm cleft phenotype. Associated diagnoses and findings were abstracted as documented in the EHR. The study did not independently re-review the underlying genetic, laboratory, imaging, or consultation reports for each entry and therefore reports documented diagnoses or findings rather than uniformly study-adjudicated diagnoses. No separate category of suspected syndrome was used; an unconfirmed suspicion would not be counted unless it appeared in the EHR as a documented diagnosis or finding. Variables were entered into a standardized, piloted data-collection form. As a quality-control exercise, a second investigator independently reviewed a random sample of 20 records using the same data dictionary and without access to the first investigator's coding. The review covered diagnosis confirmation, WHO ICD-10 mapping, analytical cleft group, laterality, and documented associated diagnoses or findings. The classifications agreed for all 20 reviewed records and variables; no adjudication was required. All five affected stillbirths had CL/P documented at birth and underwent the same EHR identification and investigator record-review process as live births. The specific clinical or postmortem method used to establish each stillbirth diagnosis was not abstracted.

### 2.6 Bias

Several potential biases were considered. Restriction to public hospitals can introduce selection bias if the case mix or population using private maternity services differs from the Dubai Health population. Tertiary services may receive a disproportionate number of complex pregnancies and neonates with associated anomalies, creating referral or case-mix effects. Retrospective EHR documentation can introduce information and misclassification bias, particularly when anatomical detail is incomplete and when complete vs. incomplete clefts cannot be distinguished reliably. Ascertainment time also differed by birth year because diagnoses recorded after discharge were eligible through March 2026. These risks were reduced through standardized query criteria, complete record review, MRN-based de-duplication, and independent quality review of 20 records, but they could not be eliminated.

### 2.7 Study size

The study used a census design and included all eligible births recorded in the participating EHR during the eight-year period. No *a priori* sample-size calculation was undertaken. Statistical precision was determined by the number of observed cases, and small subgroup counts limited power for comparisons.

### 2.8 Statistical analysis

Analyses were performed using IBM SPSS Statistics, version 31. Cross-tabulations and exact tests were computed using CROSSTABS with the Exact Tests module, and Poisson regression used GENLIN. There were no missing data for any principal variable among the 97 cases. Values documented as unspecified, including three CLP-NOS records without a recorded side, were treated as recorded categories rather than as missing. Birth prevalence was calculated as affected births divided by total births, including live births and stillbirths, and expressed per 1,000 total births. Exact Poisson 95% CIs were calculated for overall and annual rates. Categorical variables were summarized as counts and percentages. Continuous variables were summarized using means and standard deviations and, where appropriate, medians, interquartile ranges, and ranges.

Analytical cleft-group distributions among affected cases were compared across categorical characteristics using Pearson's chi-square test when assumptions were met, Fisher's exact test for small 2 × 2 tables, or Fisher-Freeman-Halton tests estimated by Monte Carlo simulation for larger sparse tables. The sex analysis used 100,000 Monte Carlo samples; the other simulated tests used 10,000. Simulation-based inference was selected because many expected cell counts were below five (27). The Monte Carlo procedure estimates a conditional exact *p*-value by sampling contingency tables with the observed margins; the resulting *p*-value is therefore subject to simulation error. No adjustment was made for multiple comparisons. All subgroup analyses were exploratory and describe cleft-group distribution among cases; they do not estimate the risk of CL/P because complete birth denominators were unavailable for the examined covariates.

Temporal change was assessed using Poisson regression with annual case count as the outcome, calendar year as a continuous predictor centered at 2018, and the logarithm of annual total births as the offset. The exponentiated year coefficient was reported as the rate ratio (RR) per calendar year with a 95% CI. Statistical significance was set at two-sided *p* < 0.05. Pearson and deviance dispersion statistics were examined. Because the series contained only eight annual observations, non-linear and changepoint models were not fitted. A documentation-based sensitivity analysis restricted the numerator to cases without a documented associated diagnosis or finding. Annual aggregate birth denominators for the three-hospital network were available, but hospital-specific denominators and the annual live-birth/stillbirth composition were not. A conventional live-birth-restricted prevalence could therefore not be calculated. We instead evaluated the expression 1,000 × 92/(46,418−S), where S is the unknown number of stillbirths, under explicitly stated scenarios. Imputation was not required because no principal data were missing.

### 2.9 Ethics

The Mohammed Bin Rashid University Institutional Review Board approved the study on 27 December 2024 (MBRU IRB-2024-627), and the Dubai Scientific Research Ethics Committee approved it on 14 January 2025 (DSREC-SR-01/2025_03). The approval and waiver of informed consent covered retrospective medical record review for the entire study period from 1 January 2018 through 31 December 2025.

## 3 Results

### 3.1 Overall birth prevalence

Across 46,418 total births recorded during 2018–2025, 97 unique CL/P cases were identified from 100 extracted rows after removal of three duplicate MRN records. The aggregate hospital-based birth prevalence was 2.09 per 1,000 total births (approximately 1 affected birth per 478 births; exact 95% CI 1.69–2.55) (Table 1). No principal case variable was missing.

**Table 1:** Annual hospital-based birth prevalence of cleft lip and/or palate in dubai health public hospitals.

Year	Total births/year	Cases, *n*	Prevalence per 1,000	Exact 95% CI
2018	6,821	13	1.91	1.01–3.26
2019	6,428	7	1.09	0.44–2.24
2020	5,654	10	1.77	0.85–3.25
2021	5,880	15	2.55	1.43–4.21
2022	5,707	14	2.45	1.34–4.12
2023	5,440	15	2.76	1.54–4.55
2024	5,315	8	1.51	0.65–2.97
2025	5,173	15	2.90	1.62–4.78
**Total**	**46,418**	**97**	**2**.**09**	**1.69–2.55**

Poisson regression with log(total births) as the offset: RR per year 1.07 (95% CI 0.98–1.17), *p* = 0.122.

### 3.2 Analytical cleft-group distribution

CP was the most frequent analytical group (48/97, 49.5%) and had a birth prevalence of 1.03 per 1,000 total births (95% CI 0.76–1.37). CL accounted for 22 cases (22.7%; 0.47 per 1,000; 95% CI 0.30–0.72): 18 were unilateral (16 left-sided and two right-sided), three were bilateral, and one was of unspecified laterality under Q36.9. UCLP/CLP-NOS accounted for 19 cases (19.6%; 0.41 per 1,000; 95% CI 0.25–0.64): 13 had documented left-sided involvement, three had documented right-sided involvement, and three were recorded as CLP NOS without a documented side. BCLP accounted for eight cases (8.2%; 0.17 per 1,000; 95% CI 0.07–0.34) (Table 2). Q35.5 was the most frequently assigned subcategory (31 cases, 32.0%). Eleven cases (11.3%) were Q35.9. Together with 27 cases assigned to Q37.8 or Q37.9, 38 of 97 cases (39.2%) lacked documentation sufficient for a more anatomically specific ICD-10 category (Supplementary Table S1).

**Table 2:** Analytical cleft-group distribution and hospital-based birth prevalence.

Mutually exclusive analytical cleft group	Cases, *n* (%)	Prevalence per 1,000 total births	Exact 95% CI
Cleft palate without lip involvement	48 (49.5)	1.03	0.76–1.37
Cleft lip without palate involvement	22 (22.7)	0.47	0.30–0.72
Unilateral cleft lip and palate or CLP NOS	19 (19.6)	0.41	0.25–0.64
Bilateral cleft lip and palate	8 (8.2)	0.17	0.07–0.34
**Total**	**97** (**100)**	**2**.**09**	**1.69**–**2.55**

### 3.3 Temporal pattern

Annual rates ranged from 1.09 per 1,000 total births in 2019 to 2.90 per 1,000 in 2025 (Table 1; Figure 1). The Poisson model did not detect a statistically significant linear temporal trend (RR per year 1.07, 95% CI 0.98–1.17; *p* = 0.122). Pearson and deviance dispersion ratios were 0.94 and 1.02, respectively, on six residual degrees of freedom, providing no evidence of material overdispersion in this eight-year aggregate series; however, these diagnostics are imprecise with only eight observations. Annual case counts ranged from seven to 15 (Figure 2).

**Figure 1:**
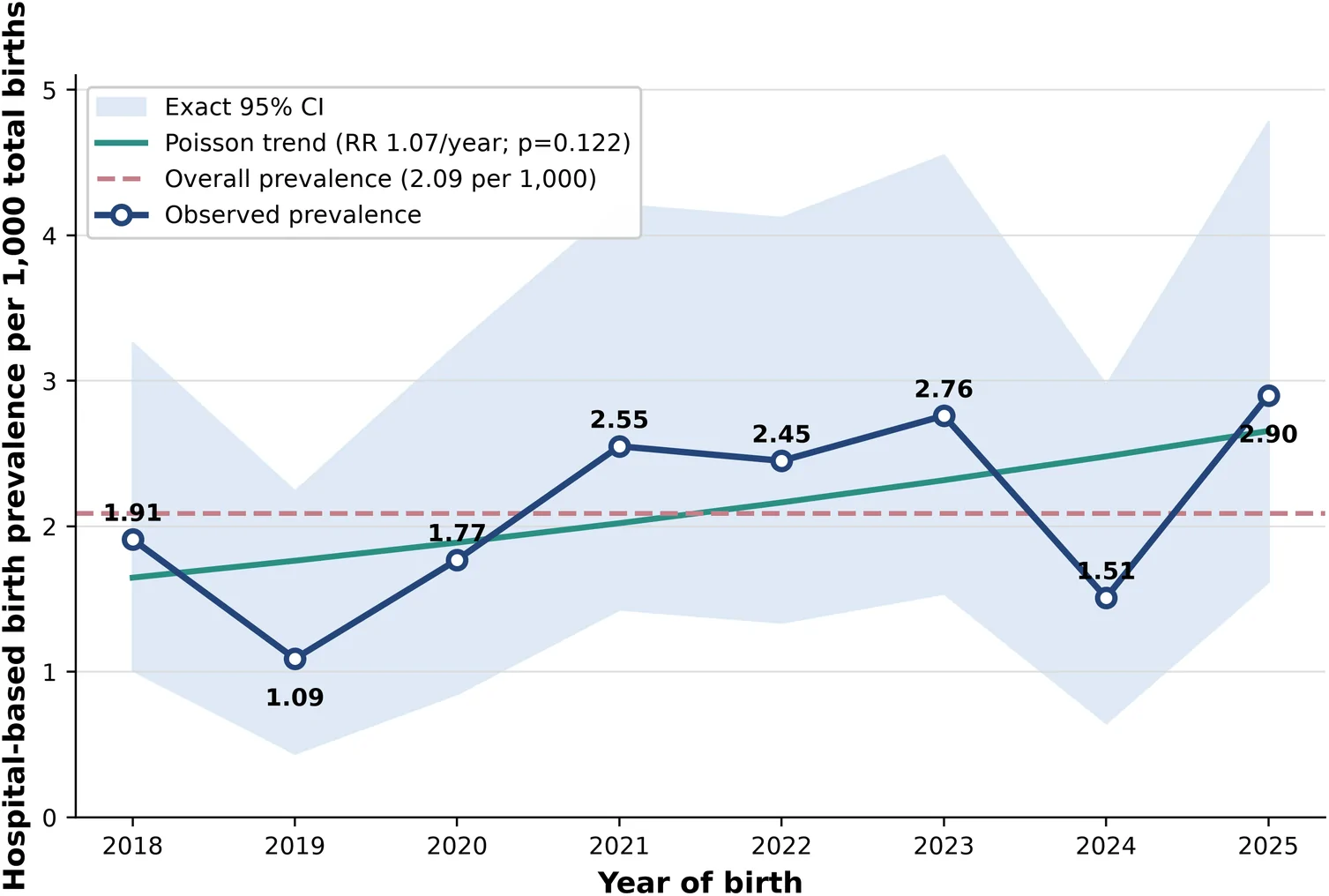
Annual birth prevalence of cleft lip and/or palate.

**Figure 2:**
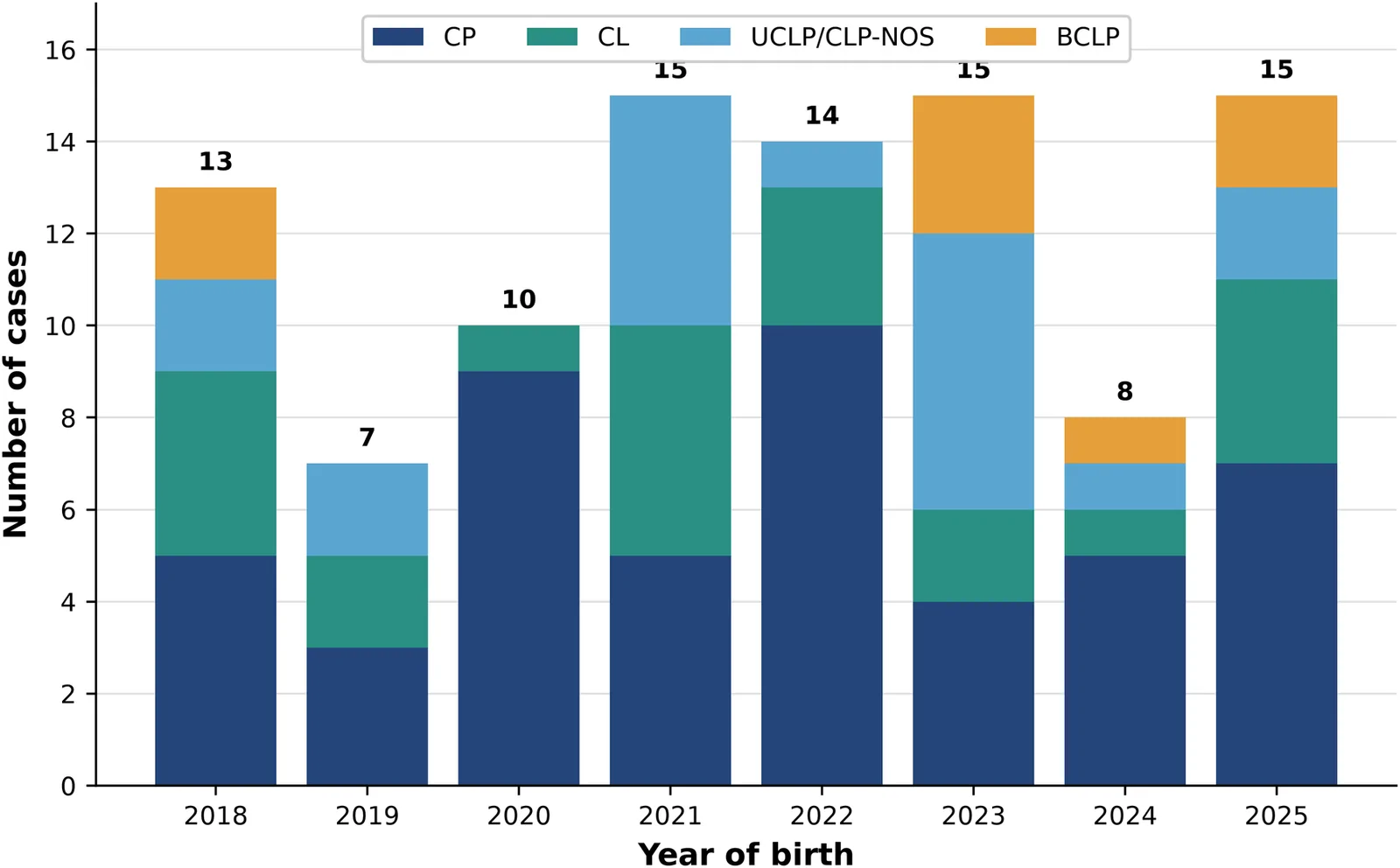
Annual case distribution by analytical cleft group.

### 3.4 Sex distribution

Among affected cases, 51 (52.6%) were male and 46 (47.4%) were female. CP was more frequent among female cases (26/48, 54.2%), whereas CL (14/22, 63.6%) and BCLP (5/8, 62.5%) were more frequent among male cases. The UCLP/CLP-NOS group included 10 males and nine females. Cleft-group distribution did not differ significantly by sex (Fisher–Freeman–Halton Monte Carlo estimate, *p* = 0.537) (Figure 3).

**Figure 3:**
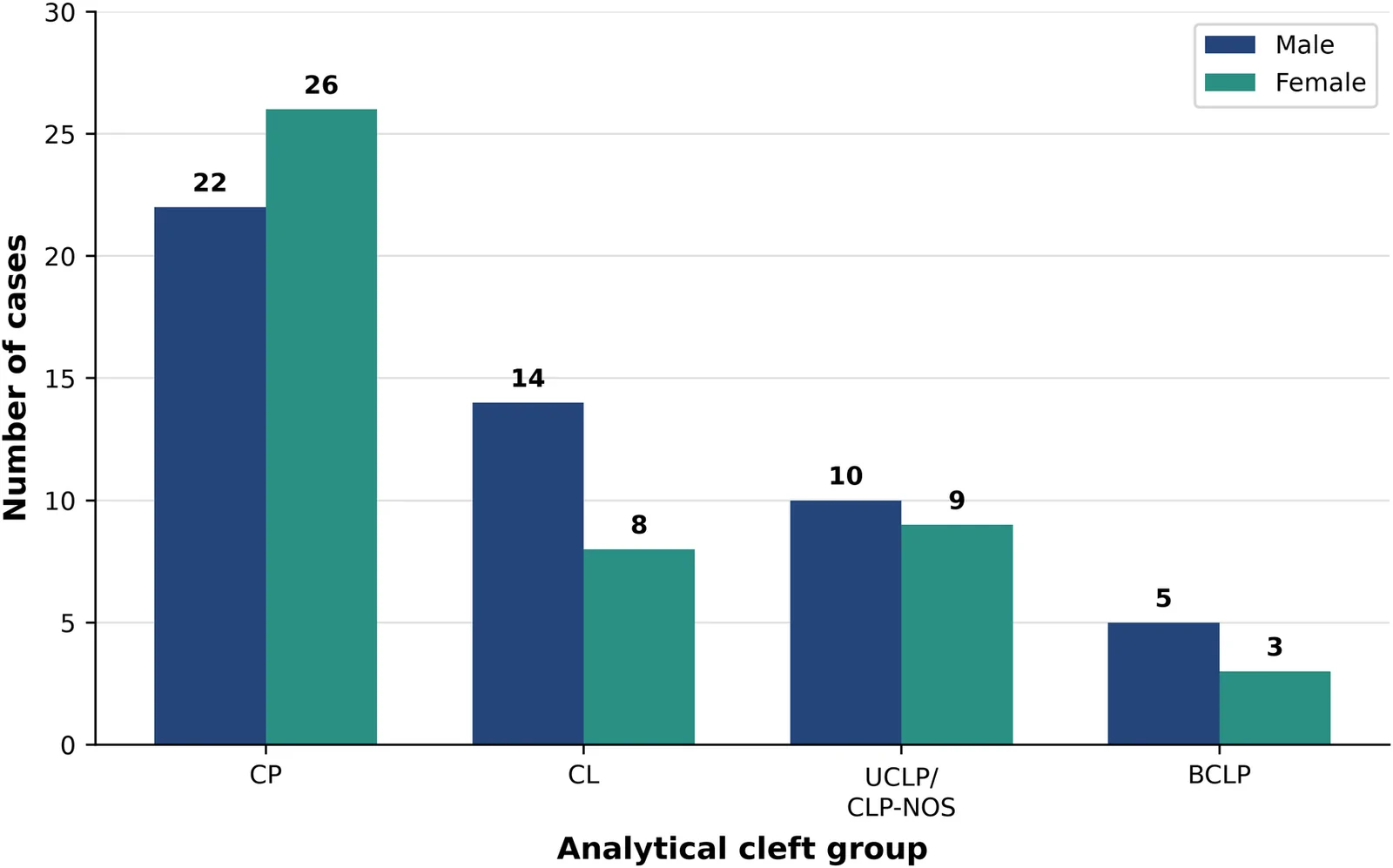
Sex distribution among cleft cases by analytical group.

### 3.5 Gestational age

Mean gestational age was 37.0 weeks (SD 3.0). Sixty-four cases (66.0%) were born at term, 32 (33.0%) were preterm, and one (1.0%) was post-term. Preterm cases comprised 19 late-preterm, six moderately preterm, and seven very-preterm births; no extremely preterm cases were recorded. Cleft-group distribution did not differ significantly across gestational-age categories (Fisher–Freeman–Halton Monte Carlo estimate, *p* = 0.223). CP accounted for five of six moderately preterm cases (83.3%), five of seven very-preterm cases (71.4%), and 27 of 64 term cases (42.2%) (Figure 4A).

**Figure 4:**
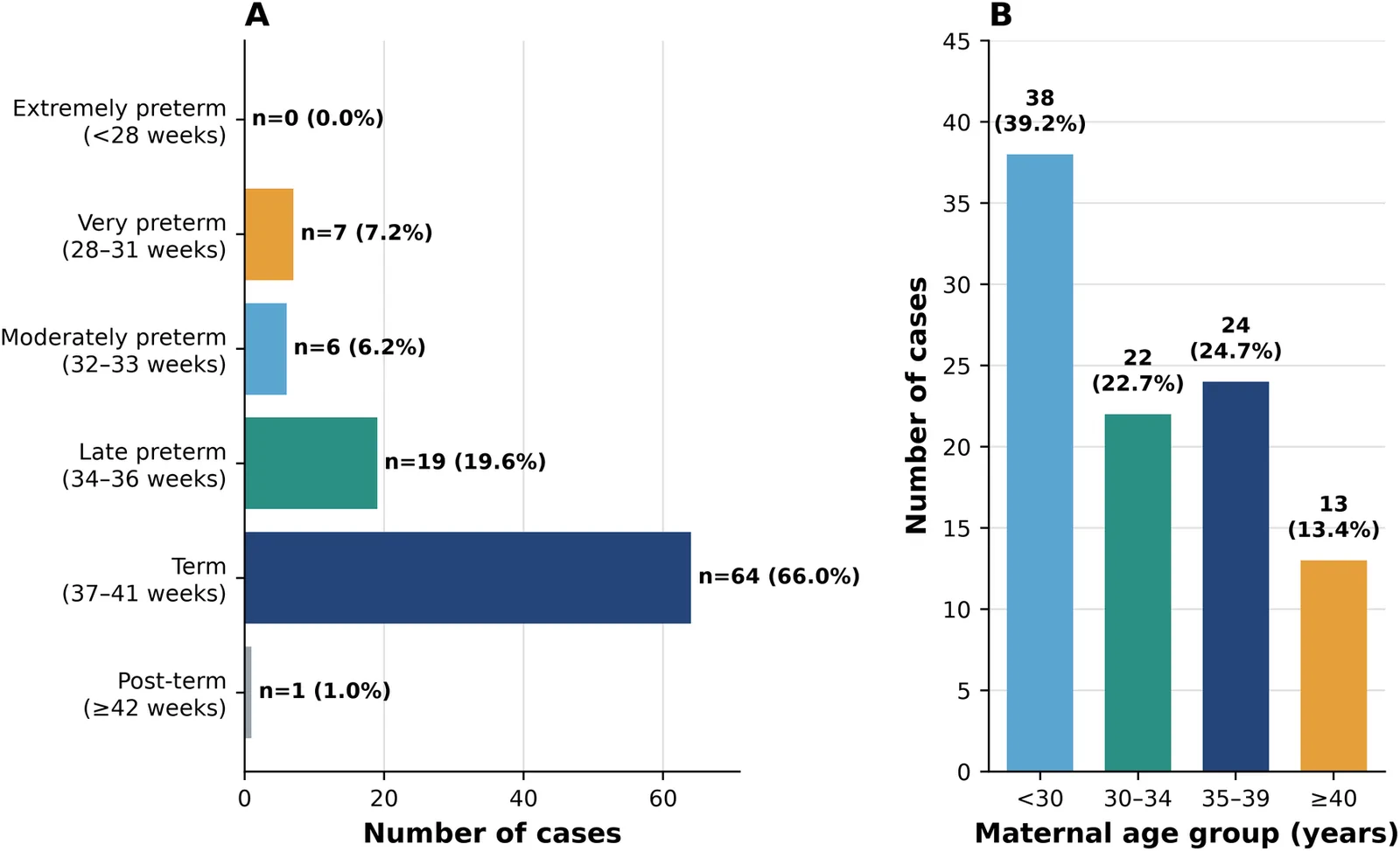
Perinatal characteristics among cleft cases. **(A)** shows gestational-age categories; **(B)** shows maternal-age categories.

### 3.6 Maternal age

Mean maternal age was 32.2 years (SD 5.9; median 31.0; interquartile range 28.0–37.0; range 19–45). Thirty-eight mothers (39.2%) were younger than 30 years, 22 (22.7%) were 30–34 years, 24 (24.7%) were 35–39 years, and 13 (13.4%) were aged 40 years or older (Figure 4B). Cleft-group distribution did not differ significantly across maternal-age categories (Fisher–Freeman–Halton Monte Carlo estimate, *p* = 0.436).

### 3.7 Nationality

The 97 cases represented 19 nationalities (Table 3). UAE nationals formed the largest case group (54, 55.7%), followed by Pakistani nationals (12, 12.4%). UAE and Omani nationals accounted for 57 cases (58.8%); South and Southeast Asian nationalities for 23 (23.7%); non-Gulf Arab nationalities for 10 (10.3%); and other nationalities for seven (7.2%). Cleft-group distribution did not differ significantly across grouped nationalities (Fisher-Freeman-Halton Monte Carlo estimate, *p* = 0.385).

**Table 3:** Nationality distribution of cleft cases.

Nationality	*n* (%)	Descriptive regional group
United Arab Emirates	54 (55.7)	Gulf Arab
Pakistan	12 (12.4)	South Asian
Philippines	4 (4.1)	Southeast Asian
India	4 (4.1)	South Asian
Yemen	3 (3.1)	Arab
Oman	3 (3.1)	Gulf Arab
Egypt	2 (2.1)	Arab
Bangladesh	2 (2.1)	South Asian
Ethiopia	2 (2.1)	Sub-Saharan African
Comoros	2 (2.1)	Sub-Saharan African
Morocco	1 (1.0)	Arab
Libya	1 (1.0)	Arab
Russia	1 (1.0)	Eastern European
Sri Lanka	1 (1.0)	South Asian
Jordan	1 (1.0)	Arab
Syria	1 (1.0)	Arab
Sudan	1 (1.0)	Sub-Saharan African
Iraq	1 (1.0)	Arab
Kyrgyzstan	1 (1.0)	Central Asian

### 3.8 Hospital and living status at birth

Most affected births were recorded at Latifa Hospital (67/97, 69.1%), followed by Dubai Hospital (27/97, 27.8%) and Hatta Hospital (3/97, 3.1%). Ninety-two cases (94.8%) were live births and five (5.2%) were stillbirths (Table 4). Because the number of stillbirths in the denominator was unavailable, live-birth-restricted prevalence could not be identified. If S denotes the unknown number of stillbirths among 46,418 total births, the corresponding live-birth prevalence is 1,000 × 92/(46,418−S). It equals 1.98 per 1,000 at the logical minimum S = 5 and 2.02 per 1,000 under an illustrative assumption that 2% of total births were stillbirths. These are scenario values, not data-derived bounds. A stillbirth proportion of 5.15% would make the live-birth-restricted estimate equal to 2.09 per 1,000. The impact of denominator composition cannot be quantified without the unavailable live-birth/stillbirth split. Cleft-group distribution did not differ significantly by hospital (Fisher–Freeman–Halton Monte Carlo estimate, *p* = 0.243) or living status at birth (Fisher-Freeman-Halton Monte Carlo estimate, *p* = 0.445).

**Table 4:** Distribution by participating hospital and living status at birth.

Variable	*n* (%)
Participating hospital	
Latifa Hospital	67 (69.1)
Dubai Hospital	27 (27.8)
Hatta Hospital	3 (3.1)
Living status at birth	
Live birth	92 (94.8)
Stillbirth	5 (5.2)

### 3.9 Documented associated diagnoses and findings

Twenty-nine cases (29.9%) had a documented associated diagnosis or finding, including a recognized syndrome, chromosomal or molecular entry, sequence, or additional congenital anomaly; 68 (70.1%) had no such diagnosis or finding documented (Table 5). Restricting the numerator to the latter 68 cases yielded 1.46 per 1,000 total births (95% CI 1.14–1.86). This documentation-based sensitivity estimate should not be interpreted as the birth prevalence of genetically or clinically confirmed nonsyndromic CL/P. The proportion with a documented associated diagnosis or finding was highest in BCLP (4/8, 50.0%), followed by CL (8/22, 36.4%), UCLP/CLP-NOS (6/19, 31.6%), and CP (11/48, 22.9%). The association between cleft group and documentation status was not statistically significant (Pearson *χ*^2^ = 3.123, df = 3; *p* = 0.373).

**Table 5:** Documented associated diagnoses or findings by analytical cleft group.

Analytical cleft group	No documented associated diagnosis or finding, *n* (%)	Documented associated diagnosis or finding, *n* (%)
Cleft palate without lip involvement	37 (77.1)	11 (22.9)
Cleft lip without palate involvement	14 (63.6)	8 (36.4)
UCLP/CLP-NOS	13 (68.4)	6 (31.6)
Bilateral cleft lip and palate	4 (50.0)	4 (50.0)
**Total**	**68** (**70.1)**	**29** (**29.9)**

Pearson *χ*^2^ = 3.123, df = 3; *p* = 0.373.

Among the 29 cases with documented associated diagnoses or findings, Robin sequence was most frequent (6/29, 20.7%), followed by Patau syndrome/trisomy 13 (5/29, 17.2%) and Dandy–Walker syndrome as documented (3/29, 10.3%). Down syndrome/trisomy 21, ring chromosome 18, and Edwards syndrome/trisomy 18 each occurred in two cases; nine other entries occurred once (Table 6). Entries were retained as documented in the EHR. The underlying laboratory, imaging, and consultation reports were not independently re-reviewed, so the table does not imply uniform diagnostic confirmation or causal relationships with the cleft phenotype. All subgroup tests and *p*-values are summarized in Table 7. No diagnosis of Stickler syndrome or 22q11.2 deletion syndrome was documented among the six Robin sequence records. These conditions were not systematically evaluated for this study, and absence of documentation should not be interpreted as their exclusion.

**Table 6:** EHR-documented associated diagnoses and findings among 29 cases.

EHR-documented diagnosis or finding	*n* (%)	Neutral classification for this report
Robin sequence	6 (20.7)	Sequence; all six had cleft palate without lip involvement
Patau syndrome/trisomy 13	5 (17.2)	Documented chromosomal diagnosis
Dandy–Walker syndrome (as documented)	3 (10.3)	Documented central nervous system malformation/syndrome label
Down syndrome/trisomy 21	2 (6.9)	Documented chromosomal diagnosis
Ring chromosome 18	2 (6.9)	Documented structural chromosomal diagnosis
Edwards syndrome/trisomy 18	2 (6.9)	Documented chromosomal diagnosis
Tuberous sclerosis	1 (3.4)	Documented multisystem genetic disorder
Chromosome 9p deletion syndrome (Alfi syndrome)	1 (3.4)	Documented chromosomal deletion syndrome
Swyer syndrome/46,XY gonadal dysgenesis	1 (3.4)	Documented disorder of sex development
Osteogenesis imperfecta	1 (3.4)	Documented heritable skeletal disorder
Skeletal dysplasia	1 (3.4)	Documented skeletal disorder
Bohring–Opitz syndrome	1 (3.4)	Documented clinical genetic syndrome
Heterozygous *TRAPPC12* variant	1 (3.4)	Molecular finding; clinical significance not established in this study
Hypoplastic left heart syndrome	1 (3.4)	Additional congenital cardiac anomaly
Potter sequence	1 (3.4)	Renal agenesis/oligohydramnios sequence

**Table 7:** Exploratory comparisons of analytical cleft-group distribution across case characteristics.

Case characteristic	Categories compared	Cases, *n*	Test applied	*p*
Sex recorded at birth	2	97	Fisher-Freeman-Halton, Monte Carlo (100,000 samples)	0.537
Gestational-age category	5	97	Fisher-Freeman-Halton, Monte Carlo (10,000 samples)	0.223
Maternal-age category	4	97	Fisher-Freeman-Halton, Monte Carlo (10,000 samples)	0.436
Nationality group	4	97	Fisher-Freeman-Halton, Monte Carlo (10,000 samples)	0.385
Birth hospital	3	97	Fisher-Freeman-Halton, Monte Carlo (10,000 samples)	0.243
Living status at birth	2	97	Fisher-Freeman-Halton, Monte Carlo (10,000 samples)	0.445
Documented associated diagnosis or finding	2	97	Pearson chi-square (*χ*^2^ = 3.123, df = 3)	0.373

## 4 Discussion

### 4.1 Principal findings and interpretation of the prevalence estimate

This eight-year study identified 97 unique CL/P cases among 46,418 births recorded in Dubai Health public hospitals, corresponding to a hospital-based birth prevalence of 2.09 per 1,000 total births. CP comprised almost half of cases, and approximately three in ten had a documented associated diagnosis or finding. Annual prevalence fluctuated, but no statistically significant linear temporal trend was detected.

The observed rate is higher than the approximately 1.1 per 1,000 live-birth average reported in the recent review of Arab-country studies and the 1.00 per 1,000 live-birth estimate from Qatar (14, 15). It is also nearly four times the 0.54 per 1,000 live births reported from Corniche Hospital in Abu Dhabi in the 1990s (16). These comparisons require caution. The present denominator includes stillbirths, whereas the Qatar and historical Abu Dhabi estimates were restricted to live births. The current study covers three public hospitals with tertiary maternity and neonatal services rather than all maternity facilities in Dubai. Differences in time period, case definitions, coding systems, diagnostic intensity, migration patterns, and referral pathways could produce substantial variation without implying a true biological difference in population risk (14, 17).

Inclusion of five affected stillbirths broadens case ascertainment relative to live-birth-only studies, but the unknown live-birth/stillbirth denominator split prevents a direct live-birth estimate. Exclusion of private-sector births also limits coverage and could shift the estimate in either direction. Minsart et al. reported 12 EUROCAT-classified isolated cleft lip/palate cases among 9,640 live births and stillbirths at ≥28 weeks in three private tertiary maternity centers in Dubai from January 2019 through July 2023, equivalent to 1.25 per 1,000 (95% CI 0.71–2.19) (28). The documentation-based sensitivity estimate of 1.46 per 1,000 in cases without a documented associated diagnosis or finding is also not directly comparable because associated diagnoses and genetic testing were not ascertained uniformly. Neither dataset can determine the reason for the numerical difference. During 2019–2024, the participating hospitals recorded 34,424 total births. The Dubai Health Statistical Yearbook reports 203,592 live births in Dubai over the same years; the hospital count is approximately 17% of that live-birth series, but this is not an exact coverage estimate because the study denominator includes stillbirths (29). Comparable emirate-wide total-birth denominators were unavailable for 2018 and 2025. The 2.09-per-1,000 estimate must therefore be interpreted as hospital-based rather than emirate-wide.

The estimate lies within the broad 0.3–2.6-per-1,000 range reported across Arab-country studies (14). Its position within that range supports standardized surveillance rather than a specific etiological explanation. Dubai's population includes many nationalities, but case composition cannot determine whether ancestry, consanguinity, migration, or other demographic factors altered prevalence. Those questions require denominator-linked designs with individual-level exposures. The genetic architecture of CL/P is complex, and regional consanguinity may be relevant to selected genetic or multifactorial presentations, but it was not measured here (4, 30–34).

The 2026 multicountry study by Alamoudi et al. provides complementary, but not directly comparable, UAE evidence (18). It described the phenotype and severity among 836 children with non-syndromic cleft lip, with or without palate, recruited from referral centers in seven countries between November 2020 and November 2021. Only 15 participants were from the UAE; the participating UAE center was outside Dubai; isolated CP was excluded from the analytic cohort; and no birth denominator was available. Its convenience sample of patients aged 0–18 years is potentially influenced by referral pathways, survival, access to cleft care, age distribution, and center participation.

Alamoudi et al. observed that severe CL/CLP phenotypes were more frequently observed in participating centers in high-income countries, but the authors appropriately acknowledged selection bias and the absence of a non-cleft comparison group (18). The finding, therefore, describes the case mix reaching those centers and should not be interpreted as a national prevalence gradient. The present study addresses a different question by enumerating CP, CL, UCLP, and BCLP among births within a defined public-hospital denominator over eight years. Together, the studies show the value of linking referral-center phenotypic detail to birth-denominator surveillance: one approach characterizes patients receiving cleft services, while the other measures occurrence within a specified birth network.

### 4.2 Analytical cleft-group distribution

CP was the largest analytical group (49.5%; 1.03 per 1,000), unlike many regional and international series in which CLP or cleft lip with or without palate predominates (15, 35–37). Between-study differences are sensitive to referral patterns, inclusion of associated conditions, ascertainment of submucous or soft-palate clefts, and classification practices (14, 38–41). Three descriptive features merit emphasis. First, CP accounted for 48 of 97 cases (49.5%). Second, the UCLP/CLP-NOS share was 8 of 27 cases (29.6%) at Dubai Hospital and 11 of 67 (16.4%) at Latifa Hospital. Third, CP contributed the largest number of cases with a documented associated diagnosis or finding (11/29, 37.9%) but the lowest within-group proportion (11/48, 22.9%); BCLP had the highest within-group proportion (4/8, 50.0%). All six Robin sequence diagnoses were in the CP group. These findings describe case composition; subgroup comparisons were not statistically significant and strata were small.

Tertiary neonatal referral is a plausible contributor because CP and Robin sequence can involve airway, feeding, neurologic, and chromosomal complexity (24, 40–45). Nevertheless, the present data cannot quantify referral bias or establish why CP predominated. Latifa Hospital, which recorded 67 cases, provides specialized maternity, neonatal, and high-risk pregnancy services and is co-located with Al Jalila Children's Hospital (46, 47). Dubai Hospital recorded 27 cases and Hatta Hospital three. Hospital-specific birth denominators, referral status, and catchment data were unavailable. These figures therefore describe case distribution rather than hospital-specific prevalence, and no inference about underlying occurrence or socioeconomic differences between hospital populations is possible.

Documentation specificity also warrants improvement. In 38 of 97 cases (39.2%), the clinical diagnosis did not support a more anatomically specific ICD-10 category: 11 were Q35.9 and 27 were Q37.8 or Q37.9. Narrative review provided additional laterality information but did not justify a more specific palatal category. Structured EHR fields combining ICD-10 with LAHSAL documentation would improve phenotype comparability (22, 23). The present general-purpose EHR enabled retrospective identification and review of available longitudinal records, but it was not a dedicated cleft registry. Ten years' experience with a national electronic medical record for facial anomalies demonstrates the value of structured multidisciplinary follow-up while also showing that incomplete outcome capture can persist in a purpose-built platform (48). A Dubai-wide registry should therefore include standardized phenotyping, scheduled outcome assessments, active follow-up, and data-completeness monitoring.

### 4.3 Temporal pattern

The point estimate increased by approximately 7% per year, but the 95% CI included no change and *p* = 0.122. The appropriate conclusion is that no statistically significant linear temporal trend was detected, not that prevalence definitively increased or remained constant. With only eight annual observations and seven to 15 cases per year, random variation produced wide CIs and limited power to detect modest change.

Registry studies in other settings have reported stable, increasing, or decreasing trends depending on time period, phenotype, ascertainment, prenatal diagnosis, and inclusion of pregnancy terminations (28, 49–52). A Swedish national study reported a decline in overall CL/P during 2000–2020, particularly for phenotypes involving the lip, whereas other administrative or program-based datasets have shown more modest or variable patterns (28, 49–52). Differences from Dubai cannot be attributed to a single cause because national registers provide broader, more standardized coverage than the current hospital network.

The study period overlapped the COVID-19 pandemic. Proposed biological and healthcare-access pathways have prompted investigation of maternal infection and congenital anomalies, but the evidence linking maternal SARS-CoV-2 infection specifically to CL/P remains uncertain (53). Individual infection timing, fever, medication, vaccination, healthcare access, and first-trimester exposure were unavailable, so the current data cannot assess a pandemic effect (53). Continued surveillance is justified, but causal interpretation of the non-significant annual pattern would be inappropriate.

### 4.4 Sex distribution

The small overall male predominance and female predominance within CP are consistent with patterns described in many cleft datasets (14, 34, 37, 54, 55). Differences in the timing of palatal shelf elevation and fusion have been proposed as one developmental explanation for sex variation, although the mechanisms are not fully established (34, 54, 55). In the present cohort, subtype distribution did not differ significantly by sex, and the BCLP group contained only eight cases. The observed 62.5% male proportion and 50% associated-diagnosis proportion in BCLP should therefore be treated as descriptive and hypothesis-generating.

The liability-threshold framework proposes that severe phenotypes can reflect a greater cumulative contribution of genetic and environmental susceptibility, but the current study did not measure genetic burden (4). It cannot determine whether the observed sex pattern or bilateral involvement reflects specific variants, chromosomal changes, environmental exposure, or chance. Larger denominator-linked registries are required for stable sex-specific estimates.

### 4.5 Gestational age

One-third of affected births were preterm, including 19 late-preterm, six moderately preterm, and seven very-preterm cases. Prior studies have reported associations between orofacial clefts and preterm birth, particularly in the presence of other anomalies or pregnancy complications (45, 56–58). In this cohort, CP was present in 5 of 6 moderately preterm cases and 5 of 7 very preterm cases, but the subtype-by-gestational-age test was not significant.

This pattern may reflect complex underlying conditions, airway or growth concerns, fetal compromise, or clinical decisions concerning delivery, yet none of these pathways was measured. More importantly, the study did not compare gestational-age-specific rates between affected and unaffected births. It therefore cannot conclude that CL/P increased the risk of prematurity or that prematurity caused a particular subtype. Linking to complete maternity records would allow for adjusted analyses of gestational age, multiple gestation, fetal growth, maternal disease, and associated anomalies.

### 4.6 Maternal age

The mean maternal age was 32.2 years, and 37 cases occurred among mothers aged 35 years or older. Subtype distribution did not differ significantly across the four age groups. Case-only comparisons cannot assess maternal-age-specific risk because the age distribution of all mothers delivering in the participating hospitals was not included.

Population-based evidence suggests a nonlinear and phenotype-specific relationship between parental age and orofacial clefts. A 2024 meta-analysis found higher risks of selected non-chromosomal congenital anomalies at very young and advanced maternal ages, while large-database analyses have also considered paternal age and residual confounding (59–61). Maternal age may correlate with chromosomal anomalies, diabetes, hypertension, medication exposure, and assisted reproduction, but these variables were not available in the current dataset. Future analyses should use maternal and paternal age denominators and multivariable models rather than interpreting counts among cases.

### 4.7 Nationality and the dubai context

UAE nationals accounted for 55.7% of cases, and the remaining cases represented 18 other nationalities. This diversity is clinically important for culturally responsive counseling and continuity of long-term cleft care. However, nationality is an imperfect proxy for ancestry, migration history, socioeconomic position, environmental exposure, and access to services. Without nationality-specific birth denominators, the observed counts cannot establish higher or lower prevalence in any group and should not be interpreted as evidence that particular nationalities are genetically predisposed.

The risk of ecological fallacy is substantial. Future work should link case counts to the nationality distribution of all births and collect individual-level family history, consanguinity, maternal health, folate use, tobacco exposure, medication use, environmental exposure, and socioeconomic measures. Regional evidence supports investigation of genetic and environmental contributors, but study designs must distinguish patient composition from population risk (4, 30–33, 62, 63).

### 4.8 Documented associated diagnoses and findings

The 29.9% proportion with a documented associated diagnosis or finding lies at the upper end of values described in cleft cohorts and may be compatible with tertiary case mix (42–45). Robin sequence was the most frequent entry, followed by trisomy 13 and Dandy–Walker syndrome as documented. BCLP had the highest within-group proportion, but the association with cleft group was not statistically significant and its denominator was small.

The EHR entries comprised recognized syndromes, sequences, chromosomal diagnoses, structural anomalies, and molecular findings and should not be treated as a uniform causal group. For anatomical analysis, all six Robin sequence records were assigned to CP because each documented cleft palate without lip involvement; Robin sequence remained an associated sequence, not an etiologically isolated cleft (24). Chromosome 9p deletion syndrome is the appropriate classification for the entry documented as Alfi syndrome (64). Established *TRAPPC12*-related encephalopathy is autosomal recessive and has been associated with biallelic variants; the single heterozygous *TRAPPC12* entry is therefore reported only as a molecular finding of unestablished clinical significance in this study (65). Comprehensive assessment remains important when multiple anomalies, growth restriction, neurologic findings, cardiac defects, or atypical cleft patterns are present (39, 42, 43, 45).

### 4.9 Strengths and limitations

This study provides the first eight-year CL/P birth-prevalence baseline for births recorded in Dubai Health public hospitals. Its census design covered all eligible live births and stillbirths in a standardized EHR across three facilities. The March 2026 query could identify diagnoses documented at birth or at later Dubai Health encounters. Clinical-record review supplemented free-text extraction, duplicate rows were removed by MRN, and a second investigator independently reproduced all reviewed classifications in a random 20-record sample. Exact CIs and offset-based Poisson regression accounted for rare annual events and changing aggregate birth denominators.

The limitations are important. First, the public-hospital cohort does not represent all births in Dubai, and the approximately 17% comparison with the 2019–2024 DHA live-birth series is not a like-for-like coverage estimate. Second, tertiary case mix could plausibly enrich the cohort for complex, preterm, or prenatally diagnosed cases, but referral status was unavailable and this explanation could not be tested. Third, retrospective free-text documentation and incomplete anatomical detail create information-loss and misclassification risks. Fourth, diagnoses recorded after discharge were eligible through March 2026; children born earlier had longer opportunities for late documentation than those born in 2025, potentially affecting late-recognized mild, incomplete, or submucous clefts. Diagnoses not recorded within Dubai Health by extraction were missed. Fifth, complete vs. incomplete clefts, submucous CP, and alveolar involvement could not be captured systematically. Sixth, although all five affected stillbirths had CL/P documented at birth, the specific confirmation method was not abstracted and equivalence of ascertainment with live births could not be evaluated. Seventh, the principal investigator assigned ICD-10 subcategories and analytical groups; complete agreement in an independently reviewed random sample of 20 records reduces but does not eliminate classification error. Eighth, small cells and multiple exploratory subgroup tests limit precision and increase the possibility of type II error. Ninth, etiological exposures—including family history, consanguinity, maternal disease, folate use, tobacco, medications, assisted reproduction, environmental exposure, parity, paternal age, and socioeconomic indicators—were unavailable or incomplete. Tenth, case-only analyses cannot estimate covariate-specific risk without denominator information. Eleventh, associated diagnoses and findings were not uniformly revalidated against source genetic, laboratory, imaging, or consultation reports; conditions associated with Robin sequence were not systematically assessed. Twelfth, hospital-specific birth denominators and the aggregate live-birth/stillbirth split were unavailable. Finally, pregnancy terminations and fetuses diagnosed outside participating birth records were not captured. The findings are therefore a baseline for the participating public hospitals, not a definitive population-wide estimate for Dubai or the UAE.

### 4.10 Clinical and public-health implications

The principal implication is the need for integrated congenital-anomaly surveillance across public and private maternity services in Dubai and, ideally, throughout the UAE. A registry should use standardized case definitions, mutually exclusive anatomical groups, structured fields for laterality and severity, and linkage to live-birth, stillbirth, termination, and validated denominator data. It should also link to genetics, surgery, audiology, speech, pediatric dentistry, orthodontics, and long-term outcomes. Such a system would help distinguish epidemiological change from shifts in referral, coding, or population composition (14, 15, 19, 22, 23, 48).

Clinical pathways should begin before or at birth when diagnosis is available and continue through adulthood. Coordinated care should include airway and feeding assessment, newborn hearing evaluation, surgical planning, speech and language follow-up, pediatric dental attendance, orthodontic review, genetic counseling when indicated, psychosocial support, and patient- and family-reported outcomes (13, 39, 66–72). The observed network average of approximately 12 affected births per year indicates sustained service demand, but this study did not measure utilization or establish differential need by cleft group. Longitudinal evidence from 166 patients with cleft lip with or without palate followed to a mean age of approximately 18 years showed that greater preoperative severity was associated with a higher cumulative number of cleft-related operations (73). Although that single-centre surgical cohort excluded isolated CP and is not directly transferable to this birth cohort, it supports linking surveillance to long-term service-planning data.

Prevention claims should remain evidence-based. The UAE introduced mandatory premarital genetic testing for Emiratis in January 2025, covering 570 genes associated with more than 840 conditions (74). This program may improve carrier identification and informed reproductive counseling for included disorders, but its effect on CL/P prevalence cannot be inferred from the current data, and the specific relevance of its panel to multifactorial non-syndromic clefts requires separate evaluation. Likewise, enhanced prenatal imaging can improve detection and preparation for care but does not, by itself, prevent cleft formation (75, 76). Experimental fetal repair and alternative timing of surgical correction remain research questions rather than current population-prevention strategies; they should not displace the immediate priorities of surveillance, coordinated care, and long-term outcomes (77, 78).

## 5 Conclusion

Among 46,418 births recorded in three Dubai Health public hospitals during 2018–2025, 97 unique CL/P cases were identified, corresponding to a hospital-based birth prevalence of 2.09 per 1,000 total births. CP was the most frequent analytical group, and 29.9% of cases had a documented associated diagnosis or finding. No statistically significant linear temporal trend or cleft-group distribution difference across the examined case characteristics was detected.

The findings establish a hospital-based baseline for the participating public hospitals and do not provide an emirate-wide population estimate for Dubai or the UAE. Comparisons must account for stillbirth inclusion, public-sector sampling, referral case mix, and phenotype definitions. Integrated public- and private-sector surveillance is needed for population-level prevalence estimation, etiological research, service planning, and longitudinal outcome evaluation.

## Data Availability

The raw data supporting the conclusions of this article will be made available by the authors, without undue reservation.

## References

[1] Mossey PA Little J Munger RG Dixon MJ Shaw WC. Cleft lip and palate. Lancet. (2009) 374:1773–85. 10.1016/S0140-6736(09)60695-4 19747722

[2] Salari N Darvishi N Heydari M Bokaee S Darvishi F Mohammadi M. Global prevalence of cleft palate, cleft lip and cleft palate and lip: a comprehensive systematic review and meta-analysis. J Stomatol Oral Maxillofac Surg. (2022) 123:110–20. 10.1016/j.jormas.2021.05.008 34033944

[3] Mossey PA Modell B. Epidemiology of oral clefts 2012: an international perspective. Front Oral Biol. (2012) 16:1–18. 10.1159/000337464 22759666

[4] Dixon MJ Marazita ML Beaty TH Murray JC. Cleft lip and palate: understanding genetic and environmental influences. Nat Rev Genet. (2011) 12:167–78. 10.1038/nrg2933 21331089 PMC3086810

[5] Sui H Du M Chen J Yang R Shi B Huang H. The impact of maternal systemic diseases on the occurrence of cleft lip and palate in newborns: a narrative review. Front Public Health. (2025) 13:1568140. 10.3389/fpubh.2025.1568140 40823220 PMC12354372

[6] Fell M Dack K Chummun S Sandy J Wren Y Lewis S. Maternal cigarette smoking and cleft lip and palate: a systematic review and meta-analysis. Cleft Palate Craniofac J. (2022) 59:1185–200. 10.1177/10556656211040015 34569861 PMC9411693

[7] Mossey PA Shaw WC Munger RG Murray JC Murthy J Little J. Global oral health inequalities: challenges in the prevention and management of orofacial clefts and potential solutions. Adv Dent Res. (2011) 23:247–58. 10.1177/0022034511402083 21490237 PMC6699117

[8] Sabbagh HJ Mossey PA Innes NP. Prevalence of orofacial clefts in Saudi arabia and neighboring countries: a systematic review. Saudi Dent J. (2012) 24:3–10. 10.1016/j.sdentj.2011.11.001 23960521 PMC3723074

[9] Haque S Alam MK. Common dental anomalies in cleft lip and palate patients. Malays J Med Sci. (2015) 22:55–60. PubMed: PMID 26023296. 26023296 PMC4438093

[10] James O Otoghile B Adamson OO Egbunah UP Sabo VY Adekunle AA. Otological challenges in patients with non-syndromic cleft lip and palate: a systematic review. FACE. (2023) 4:281–93. 10.1177/27325016231184131

[11] Tsuchiya S Tsuchiya M Momma H Koseki T Igarashi K Nagatomi R. Association of cleft lip and palate on mother-to-infant bonding: a cross-sectional study in the Japan environment and children’s study. BMC Pediatr. (2019) 19:505. 10.1186/s12887-019-1877-9 31862001 PMC6923825

[12] Yusof MS Mohd Ibrahim H. The impact of cleft lip and palate on the quality of life of young children: a scoping review. Med J Malaysia. (2023) 78:250–8. 36988538

[13] Gigov K Ginev I Miteva M. Cleft lip and/or palate: a comprehensive review and our treatment approach. Acta Med Bulg. (2026) 53:85–92. 10.2478/amb-2026-0049

[14] Bugaighis I. A critical review of the prevalence of cleft lip and cleft palate in arab countries. East Mediterr Health J. (2025) 31:471–7. 10.26719/2025.31.7.471 40832868

[15] Khandakji MN Hammamy M Albaloushi M Alhashimi N. The prevalence of orofacial clefts in Qatar: a cross-sectional nationwide study. BMC Oral Health. (2024) 24:926. 10.1186/s12903-024-04455-8 39127631 PMC11316983

[16] Al Talabani J Shubbar AI Mustafa KE. Major congenital malformations in United Arab Emirates (UAE): need for genetic counselling. Ann Hum Genet. (1998) 62:411–8. 10.1046/j.1469-1809.1998.6250411.x 10088038

[17] Al Hosani H Saade D Zakaria M Edwards MA Czeizel AE. A comparison of infants affected with congenital abnormalities born in the inpatient clinic or treated in the outpatient clinic of the same hospital in the United Arab Emirates. Congenit Anom. (1999) 39:253–60. 10.1111/j.1741-4520.1999.tb00564.x

[18] Alamoudi RA Abudawood SN Abdulhameed FD Alnamnakani EA Rabah SM Hamdan MA. Cleft lip with or without palate in seven countries with two income levels: a cross-sectional study. BMC Oral Health. (2026) 26:1726. 10.1186/s12903-026-08689-6 42231242 PMC13573451

[19] Von Elm E Altman DG Egger M Pocock SJ Gøtzsche PC Vandenbroucke JP. The strengthening the reporting of observational studies in epidemiology statement: guidelines for reporting observational studies. Lancet. (2007) 370:1453–7. 10.1016/S0140-6736(07)61602-X 18064739

[20] Computer Weekly. Dubai Health Authority completes final phase of Salama electronic medical records system roll-out (2017). Available online at: https://www.computerweekly.com/news/450430353/Dubai-Health-Authority-completes-final-phase-of-Salama-electronic-medical-records-system-roll-out (Accessed August 28, 2026).

[21] OBIX. Dubai Health partners with OBIX to enhance maternity and newborn care (2025). Available online at: https://www.obix.com/dubai-health-partners-with-obix-to-enhance-maternity-and-newborn-care/ (Accessed August 28, 2026).

[22] World Health Organization. International Statistical Classification of Diseases and Related Health Problems, 10th Revision. Geneva: World Health Organization (2016).

[23] Kriens O. LAHSAL: a concise documentation system for cleft lip, alveolus, and palate diagnoses. In: Kriens O, editor. What is Cleft Lip and Palate? A Multidisciplinary Update. Stuttgart: Thieme (1989) p. 30–4.

[24] Breugem CC Evans KN Poets CF Suri S Picard A Filip C. Best practices for the diagnosis and evaluation of infants with robin sequence: a clinical consensus report. JAMA Pediatr. (2016) 170:894–902. 10.1001/jamapediatrics.2016.0796 27429161

[25] Goldenberg RL Culhane JF Iams JD Romero R. Epidemiology and causes of preterm birth. Lancet. (2008) 371:75–84. 10.1016/S0140-6736(08)60074-4 18177778 PMC7134569

[26] Gantt A Metz TD Kuller JA Louis JM Cahill AG Turrentine MA. Obstetric care consensus no. 11: pregnancy at age 35 years or older. Am J Obstet Gynecol. (2023) 228:B25–40. 10.1016/j.ajog.2022.07.022 35850202

[27] Agresti A. Categorical Data Analysis. 2nd ed. Hoboken, NJ: Wiley (2002).

[28] Minsart AF Adam H Ghenimi N Tahlak M Mirza FG Akbar A. Prevalence and prenatal diagnosis of major congenital anomalies in dubai: first insights and emerging patterns in a multicenter cohort study. BMC Pregnancy Childbirth. (2025) 25:806. 10.1186/s12884-025-07934-0 40750863 PMC12317602

[29] Dubai Health Authority. Dubai Health Statistical Yearbook 2024. Dubai: Dubai Health Authority (2025).

[30] Tadmouri GO Nair P Obeid T Al Ali MT Al Khaja N Hamamy HA. Consanguinity and reproductive health among arabs. Reprod Health. (2009) 6:17. 10.1186/1742-4755-6-17 19811666 PMC2765422

[31] Sabbagh HJ Hassan MH Innes NP Baik AA Mossey PA. Parental consanguinity and nonsyndromic orofacial clefts in children: a systematic review and meta-analyses. Cleft Palate Craniofac J. (2014) 51:501–13. 10.1597/12-209 23638915

[32] Elahi MM Jackson IT Elahi O Khan AH Mubarak F Tariq GB. Epidemiology of cleft lip and cleft palate in Pakistan. Plast Reconstr Surg. (2004) 113:1548–55. 10.1097/01.PRS.0000117184.77459.2B 15114113

[33] Bizzari S Nair P Hana S Deepthi A Al-Ali MT Al-Gazali L. Spectrum of genetic disorders and gene variants in the United Arab Emirates national population: insights from the CTGA database. Front Genet. (2023) 14:1177204. 10.3389/fgene.2023.1177204 37214420 PMC10194840

[34] Merritt L. Part 1. Understanding the embryology and genetics of cleft lip and palate. Adv Neonatal Care. (2005) 5:64–71. 10.1016/j.adnc.2004.12.006 15806447

[35] Albalawi F Alsaeed S Alalola B Alotaib GS Kalagi S. Prevalence and patterns of orofacial clefts among children from different regions of Saudi arabia: a systematic review. Int J Clin Pediatr Dent. (2023) 16:124–30. 10.5005/jp-journals-10005-2507 37020763 PMC10068006

[36] Sabbagh HJ Innes NP Sallout BI Alamoudi NM Hamdan MA Alhamlan N. Birth prevalence of non-syndromic orofacial clefts in Saudi arabia and the effects of parental consanguinity. Saudi Med J. (2015) 36:1076–83. 10.15537/smj.2015.9.11823 26318465 PMC4613632

[37] Yow M Jin Y Yeo HL Wee BY Yong CW Lim J. Epidemiologic trends of infants with orofacial clefts in a multiethnic country: a retrospective population-based study. Sci Rep. (2021) 11:7807. 10.1038/s41598-021-87229-4 33824370 PMC8024282

[38] Yusra Elfaidy M Eman Naas A Arij Arebi GSH Hend Alfowers NM Hadil Albourawi MA Surur Elyounsi N. Epidemiology of orofacial clefts: a spatio-temporal descriptive analysis in Tripoli, Libya, 2017–2021. Alq J Med App Sci. (2024) 7:1249–55. 10.54361/ajmas.247449

[39] Mink van der Molen AB Van Breugel JMM Janssen NG Admiraal RJC Van Adrichem LNA Bierenbroodspot F. Clinical practice guidelines on the treatment of patients with cleft lip, alveolus, and palate: an executive summary. J Clin Med. (2021) 10:4813. 10.3390/jcm10214813 34768332 PMC8584510

[40] Burg ML Chai Y Yao CA Magee W Figueiredo JC. Epidemiology, etiology, and treatment of isolated cleft palate. Front Physiol. (2016) 7:67. 10.3389/fphys.2016.00067 26973535 PMC4771933

[41] Fujii N Doi R Narai T Chiuriki N Ikuta M Kodani I. Clinical characteristics of the cleft lip and/or palate: association with congenital anomalies, syndromes, and chromosomal anomalies. Yonago Acta Med. (2023) 66:440–7. 10.33160/yam.2023.11.009 38028265 PMC10674057

[42] Bartzela T Theuerkauf B Reichardt E Spielmann M Opitz C. Clinical characterization of 266 patients and family members with cleft lip and/or palate with associated malformations and syndromes. Clin Oral Investig. (2021) 25:5531–40. 10.1007/s00784-021-03863-2 33760974 PMC8370934

[43] Mc Goldrick N Revie G Groisman B Hurtado-Villa P Sipek A Khoshnood B. A multi-program analysis of cleft lip with cleft palate prevalence and mortality using data from 22 international clearinghouse for birth defects surveillance and research programs, 1974–2014. Birth Defects Res. (2023) 115:980–97. 10.1002/bdr2.2176 37186369 PMC10405782

[44] Philipp K Anja Q Boris S Johanna K Susanne W Adam S. Epidemiological and clinical evaluation of patients with a cleft in lower saxony, Germany: a monocenter analysis. Clin Oral Investig. (2023) 27:5661–70. 10.1007/s00784-023-05187-9 37542681 PMC10492882

[45] Ludorf KL Benjamin RH Canfield MA Swartz MD Agopian AJ. Prediction of preterm birth among infants with orofacial cleft defects. Cleft Palate Craniofac J. (2025) 62:35–43. 10.1177/10556656231198945 37671412 PMC11363204

[46] Dubai Health Authority. Latifa Women & Children Hospital Service Catalogue. Dubai: Dubai Health Authority (updated 2022) (2022).

[47] Dubai Health. Latifa Hospital: specialized care for women (2026). Available online at: https://dubaihealth.ae/facility/hospital/latifa-hospital (Accessed September 17, 2026).

[48] Anastassov Y Ginev I Velikova R Markova Y Ivanova M Dimova G. Fourth edition of the electronic medical record for facial anomalies—ten years of experience. C R Acad Bulg Sci. (2024) 77:906–13. 10.7546/CRABS.2024.06.14

[49] Cornefjord M Källén K Klintö K Stiernman M Wiedel AP Becker M. Birth prevalence of cleft lip and/or palate: a register study of all children born in Sweden 2000–2020. J Plast Surg Hand Surg. (2025) 60:120–6. 10.2340/jphs.v60.43739 40457887

[50] Heydari M-H Sadeghian A Khadivi G Mustafa HJ Javinani A Nadjmi N. Prevalence, trend, and associated risk factors for cleft lip with/without cleft palate: a national study on live births from 2016 to 2021. BMC Oral Health. (2024) 24:36. 10.1186/s12903-023-03797-z 38185687 PMC10771673

[51] Ho J Casas M. Temporal trends in new registrations for the Ontario cleft lip and palate/craniofacial dental program between 2007 and 2018. Cleft Palate Craniofac J. (2021) 58:90–7. 10.1177/1055665620936095 32613836

[52] Taritsa IC Ledwon JK Bajaj A Gosain AK. Twelve-year trends of orofacial clefts in the United States: highlighting racial/ethnic differences in prevalence of cleft lip and cleft palate. Cleft Palate Craniofac J. (2025) 62:820–8. 10.1177/10556656241227033 38291621

[53] Joy P Kunthavai R Sahu S Routray S. COVID-19 and congenital cleft lip and palate: a systematic review focusing on nonsyndromic neonatal outcomes. Cureus. (2026) 18:e106878. 10.7759/cureus.106878 42131658 PMC13162173

[54] Murray JC. Gene/environment causes of cleft lip and/or palate. Clin Genet. (2002) 61:248–56. 10.1034/j.1399-0004.2002.610402.x 12030886

[55] Pool SMW Van Der Lek LM De Jong K Vermeij-Keers C Mouës-Vink CM. Embryologically based classification specifies sex differences in the prevalence of orofacial cleft subphenotypes. Cleft Palate Craniofac J. (2021) 58:54–60. 10.1177/1055665620935363 32602363 PMC7739112

[56] Lei RL Chen HS Huang BY Chen YC Chen PKT Lee HY. Population-based study of birth prevalence and factors associated with cleft lip and/or palate in Taiwan 2002–2009. PLoS One. (2013) 8:e58690. 10.1371/journal.pone.0058690 23555592 PMC3608649

[57] Wyszynski DF Sarkozi A Vargha P Czeizel AE. Birth weight and gestational age of newborns with cleft lip with or without cleft palate and with isolated cleft palate. J Clin Pediatr Dent. (2003) 27:185–90. 10.17796/jcpd.27.2.475367q2601u3x4w 12597694

[58] Shehan JN Danis DON Bains A Basa K Marston AP Levi JR. Cleft palate in newborns diagnosed with prematurity. Otolaryngol Head Neck Surg. (2021) 165:887–94. 10.1177/01945998211002148 33752517

[59] Pethő B Váncsa S Váradi A Agócs G Mátrai Á Zászkaliczky-Iker F. Very young and advanced maternal age strongly elevates the occurrence of nonchromosomal congenital anomalies: a systematic review and meta-analysis of population-based studies. Am J Obstet Gynecol. (2024) 231:490–500.e73. 10.1016/j.ajog.2024.05.010 38761840

[60] Ács M Cavalcante BGN Bănărescu M Wenning AS Hegyi P Szabó B. Maternal factors increase risk of orofacial cleft: a meta-analysis. Sci Rep. (2024) 14:28104. 10.1038/s41598-024-79346-7 PMC11568291 39548204

[61] Thota B Kang AW Krakauer K Tran H-V Grande P Shah J. A nationwide analysis of the association between parental age and incidence of cleft lip/palate. J Plast Reconstr Aesthet Surg. (2025) 104:328–33. 10.1016/j.bjps.2025.02.050 40158409

[62] Hamamy H. Consanguineous marriages: preconception consultation in primary health care settings. J Community Genet. (2012) 3:185–92. 10.1007/s12687-011-0072-y 22109912 PMC3419292

[63] Musaad Alghamdi S Johar Aljohar A Abdullah Alamoudi R Sulaiman Alrejaye N Dawood Abdulhameed F Mahdi Alhussain R. Assessment of non-syndromic orofacial cleft severity and associated environmental factors in Saudi arabia: a cross-sectional study. Saudi Dent J. (2024) 36:480–5. 10.1016/j.sdentj.2023.12.009 38525175 PMC10960119

[64] Starosta RT Jensen N Couteranis S Slaugh R Easterlin D Tate V. Using a new analytic approach for genotyping and phenotyping chromosome 9p deletion syndrome. Eur J Hum Genet. (2024) 32:1095–105. 10.1038/s41431-024-01667-y 38972963 PMC11369271

[65] Milev MP Grout ME Saint-Dic D Cheng Y-HH Glass IA Hale CJ. Mutations in *TRAPPC12* manifest in progressive childhood encephalopathy and Golgi dysfunction. Am J Hum Genet. (2017) 101:291–9. 10.1016/j.ajhg.2017.07.006 28777934 PMC5544387

[66] Frederick R Hogan AC Seabolt N Stocks RMS. An ideal multidisciplinary cleft lip and cleft palate care team. Oral Dis. (2022) 28:1412–7. 10.1111/odi.14213 35429208

[67] Saikia A Muthu MS Orenuga OO Mossey P Ousehal L Yan S. Systematic review of clinical practice guidelines for oral health in children with cleft lip and palate. Cleft Palate Craniofac J. (2022) 59:800–14. 10.1177/10556656211025189 34159833 PMC9121521

[68] Rabah SM Sabbagh HJ AlZabin A Almajed E Albrahim R Aldawish R. Parental knowledge and barriers to cleft lip and palate care: a cross-cultural study from the Middle East and south Asia. Front Public Health. (2026) 13:1691553. 10.3389/fpubh.2025.1691553 41584146 PMC12827703

[69] Karki S Horváth J Laitala M-L Vástyán A Nagy Á Sándor GK. Validating and assessing oral health-related quality of life among Hungarian children with cleft lip and palate using the child-OIDP scale. Eur Arch Paediatr Dent. (2021) 22:57–65. 10.1007/s40368-020-00525-x 32323224 PMC7943501

[70] Xia W Du M Wu M Chen Z Yang R Shi B. Patient-reported outcomes measure for patients with cleft palate. Front Public Health. (2024) 12:1469455. 10.3389/fpubh.2024.1469455 39281080 PMC11393830

[71] Lentge K Lentge F Zeller A-N Gellrich N-C Tavassol F Korn P. Cleft lip and palate: the psychological burden of affected parents during the first three years of their children’s lives. Int J Oral Maxillofac Surg. (2022) 51:1462–8. 10.1016/j.ijom.2022.04.003 35513959

[72] Ma Q Wei J Peng B Liu J Mo S. Burden of orofacial clefts from 1990 to 2021 at global, regional, and national levels. Front Pediatr. (2025) 13:1502877. 10.3389/fped.2025.1502877 40191646 PMC11968431

[73] Ginev I Anastassov Y Gigov K Kavradzhieva P. From severity to surgical load: long-term burden of care in cleft lip patients. Dent J (Basel). (2026) 14:376. 10.3390/dj14060376 42345966 PMC13298796

[74] Ministry of Health and Prevention, United Arab Emirates. Genetic testing as part of premarital screening for Emiratis (2024). Available online at: https://mohap.gov.ae/en/w/genetic-testing-as-part-of-premarital-screening-for-emiratis (Accessed August 28, 2026).

[75] Chedid SJ De Fantini SM Deeken G Vasanthan LT Leite HV Caldas JVJ. Antenatal ultrasound (AUS) and the detection of cranio- and orofacial malformations—a scoping review. J Public Health Emerg. (2025) 9:5. 10.21037/jphe-24-59

[76] Guichoud Y El Ezzi O De Buys Roessingh A. Cleft lip and palate antenatal diagnosis: a Swiss university center performance analysis. Diagnostics. (2023) 13:2479. 10.3390/diagnostics13152479 37568842 PMC10416856

[77] Papadopulos NA Papadopoulos MA Kovacs L Zeilhofer HF Henke J Boettcher P. Foetal surgery and cleft lip and palate: current status and new perspectives. Br J Plast Surg. (2005) 58:593–607. 10.1016/j.bjps.2005.01.014 15992528

[78] Walsh K Foy C. Surgical repair of cleft lip: comparison of neonatal and standard timing in a systematic review. Cureus. (2025) 17:e92858. 10.7759/cureus.92858 41127728 PMC12539755

