# Supplementary material for: Birth prevalence and characteristics of cleft lip and/or palate in dubai health public hospitals, 2018–2025: a retrospective hospital-based study
Source: Front Dent Med. 2026 Oct 14;7:1982859. doi: 10.3389/fdmed.2026.1982859 (PMC13713270; doi:10.3389/fdmed.2026.1982859)
Supplement: Supplementary file 1 [file Table1.docx]

**Supplementary Material**

Birth Prevalence and Characteristics of Cleft Lip and/or Palate in Dubai Health Public Hospitals, 2018–2025: A Retrospective Hospital-Based Study

**Supplementary Table S1. WHO ICD-10 subcategories assigned to the 97 cases.**

| **ICD-10 code** | **Description** | **Cases, n (%)** | **Analytical cleft group** |
| --- | --- | --- | --- |
| Q35.1 | Cleft hard palate | 1 (1.0) | Cleft palate without lip involvement |
| Q35.3 | Cleft soft palate | 5 (5.2) | Cleft palate without lip involvement |
| Q35.5 | Cleft hard palate with cleft soft palate | 31 (32.0) | Cleft palate without lip involvement |
| Q35.9 | Cleft palate, unspecified | 11 (11.3) | Cleft palate without lip involvement |
| Q36.0 | Cleft lip, bilateral | 3 (3.1) | Cleft lip without palate involvement |
| Q36.9 | Cleft lip, unilateral (includes cleft lip NOS) | 19 (19.6) | Cleft lip without palate involvement |
| Q37.8 | Unspecified cleft palate with bilateral cleft lip | 8 (8.2) | Bilateral cleft lip and palate |
| Q37.9 | Unspecified cleft palate with unilateral cleft lip (includes cleft palate with cleft lip NOS) | 19 (19.6) | UCLP/CLP-NOS |
| Total |  | 97 (100) |  |

Eight of the 16 searched ICD-10 subcategories were represented. Codes were assigned by the principal investigator from documented free-text diagnoses. In 38 cases (39.2%), comprising 11 Q35.9 and 27 Q37.8/Q37.9 records, documentation did not support a more anatomically specific ICD-10 category. Laterality was retained as unspecified when no side was documented and was not inferred from the code.

Abbreviations: ICD-10, International Classification of Diseases, 10th Revision; UCLP, unilateral cleft lip and palate; CLP-NOS, cleft lip and palate not otherwise specified.
